# Hungary as a source of West Nile virus diversity and spread in Europe: insights from the 2024 transmission season

**DOI:** 10.2807/1560-7917.ES.2026.31.16.2500785

**Published:** 2026-04-23

**Authors:** Anna Nagy, Károly Erdélyi, Zsuzsanna Molnár, Renáta Bagóné Lőrincz, Orsolya Nagy, Anita Koroknai, Nikolett Csonka, Kata Kerényi, Petra Forgách, Enikő Horváth, Zoltán Soltész, Gergely Nagy, Mária Takács, Erzsébet Barcsay, Katalin Szomor, Gábor Endre Tóth, Dániel Cadar

**Affiliations:** 1 National Reference Laboratory for Viral Zoonoses, National Center for Public Health and Pharmacy, Budapest, Hungary; 2 HUN-REN Veterinary Medical Research Institute, Budapest, Hungary; 3 Department of Microbiology and Infectious Diseases, University of Veterinary Medicine Budapest, Budapest, Hungary; 4 Health Safety National Laboratory, Budapest, Hungary; 5 Unit of Communicable Disease Epidemiology and Immunisation, National Center for Public Health and Pharmacy, Budapest, Hungary; 6 Institute of Medical Microbiology, Semmelweis University, Budapest, Hungary; 7 Institute of Ecology and Botany, HUN-REN Centre for Ecology Research, Vácrátót, Hungary; 8 National Laboratory for Health Security, HUN-REN Centre for Ecological Research, Budapest, Hungary; 9 Division of Microbiological Reference Laboratories, National Center for Public Health and Pharmacy, Budapest, Hungary; 10 Virus Metagenomics and Evolution Group, Department of Arbovirology and Entomology, Bernhard Nocht Institute for Tropical Medicine, Hamburg, Germany

**Keywords:** Virus genomics, West Nile virus, phylogeography, Hungary, surveillance, One Health

## Abstract

**BACKGROUND:**

West Nile virus (WNV) has become established across Europe, with Hungary serving as a key transmission hub since 2004. Following reduced activity during 2020–22, the 2024 season marked a resurgence with the largest geographical distribution ever recorded in Europe.

**AIM:**

To analyse the 2024 WNV transmission season in Hungary using a One Health approach and characterise circulating strains within the European phylogeographic context using comprehensive genomic surveillance.

**METHODS:**

Complete and near-complete genome sequencing was performed on 55 specimens from 38 humans, 15 birds and two Culex pipiens mosquito pools using amplicon-based next-generation sequencing. Phylogeographic analysis incorporated 637 European WNV genome sequences (2004–24) with time-scaled Bayesian phylogenetic reconstruction and continuous spatial diffusion modelling.

**RESULTS:**

Hungary reported 113 human WNV cases in 2024 (n = 111 autochthonous, 2 imported), a 3.7-fold increase from 2023 (incidence: 1.16 vs 0.31 per 100,000 population). Neuroinvasive disease predominated (92%, n = 104) with a 7.9% case fatality rate. All 55 sequenced strains belonged to WNV lineage 2. Phylogeographic analysis revealed Hungary's central role in European WNV dissemination since 2004, with multiple introductions and local diversification across distinct clades. Continuous spatial modelling identified Hungary as a persistent transmission hub with bidirectional viral flow to neighbouring countries, contributing to northward expansion.

**CONCLUSION:**

Hungary remains a critical WNV transmission hub in Central Europe with established endemicity of multiple lineage 2 clades. The analysis highlights Hungary's role as both a recipient and major source of European WNV diversity, emphasising the need for coordinated surveillance and climate-adapted preparedness strategies.

**Key public health message**

**What did you want to address in this study and why?**

Hungary is at the crossroads of major bird migration routes and has many wetlands that favour mosquito breeding, making it a natural hotspot for West Nile virus (WNV) circulation. We wanted to understand why Hungary was one of the most affected countries during the 2024 WNV season. By combining human, animal and mosquito data with genomic analysis of the virus, we aimed to clarify how WNV spreads within Hungary and across Europe.

**What have we learnt from this study?**

Hungary's 2024 WNV outbreak was the country's second largest, with 113 confirmed human cases. Genetic analyses revealed greater WNV diversity than in any other European country, establishing it as a key source and amplifier of continental transmission. Spread followed two main routes, westward into Austria and Central Europe, and southward into the Balkans and Greece, with occasional long-distance dispersal to Italy and Spain.

**What are the implications of your findings for public health?**

Our findings show that Hungary is a central hub for WNV spread in Europe, necessitating stronger mosquito surveillance, better clinical awareness and integrated ‘One Health’ monitoring. As climate change drives warmer temperatures and longer mosquito seasons, the risk of larger and earlier outbreaks will increase. Coordinated European surveillance and preparedness are critical to protect public health and prevent future epidemics.

## Introduction

West Nile virus (WNV, *Orthoflavivirus nilense*), an orthoflavivirus of the family Flaviviridae, is maintained in an enzootic cycle between ornithophilic mosquitoes (primarily Culex species) and birds, with humans serving as incidental dead-end hosts [1]. Approximately 80% of human infections remain asymptomatic, 20% develop West Nile fever (WNF) and less than 1% progress to severe West Nile neuroinvasive disease (WNND), which carries substantial case fatality, particularly in elderly (> 60 years) and immunocompromised individuals [2].

Since its European emergence in south-eastern regions during the 20th century, WNV has demonstrated sustained northward expansion, with lineages 1 and 2 now endemic across multiple countries [3]. The 2018 season recorded the largest European outbreak with 2,115 reported human cases, establishing WNV as a major public health concern across the continent [4]. Following a period of reduced activity during the COVID-19 pandemic (2020–23), WNV transmission resurged markedly in 2024. A total of 19 European countries reported 1,436 autochthonous cases across 212 regions, representing the largest geographical distribution ever recorded, and surpassing the number of affected areas (n  =  173) in 2018 [5].

Hungary occupies a unique geographical position in European WNV epidemiology: the first lineage 2 strain of WNV (WNV-L2) was detected in 2004 and thereafter, the country became a major transmission hub in Central Europe [3]. The country's strategic location at the intersection of major migratory bird flyways, combined with extensive wetland systems including the Tisza and Danube River basins and Lake Balaton region, creates optimal ecological conditions for WNV maintenance and dispersal [6]. Since 2004, Hungary has consistently reported WNV cases, with notable peaks in 2008–09 and 2018, reflecting broader European epidemic patterns. In addition to WNV, Hungary hosts other endemic orthoflaviviruses including tick-borne encephalitis virus (TBEV) and Usutu virus (USUV), creating complex diagnostic challenges caused by serological cross-reactivity among closely related viruses within the same antigenic complex [7]. This epidemiological complexity requires sophisticated laboratory algorithms and emphasises the importance of molecular characterisation for accurate strain identification and phylogenetic analysis.

The European Centre for Disease Prevention and Control (ECDC) coordinates continental WNV surveillance through the European surveillance portal for infectious diseases (EpiPulse), enabling real-time monitoring of transmission patterns in humans and facilitating a rapid response to emerging threats [8]. This coordinated approach has been crucial for understanding the transboundary nature of WNV transmission and informing evidence-based control strategies. By applying an integrated One Health approach that combines human surveillance data with animal and environmental data, we aimed to provide a comprehensive analysis of the 2024 WNV transmission season in Hungary using phylogeographic analysis. We characterised circulating WNV strains within the broader European context using time-scaled Bayesian phylogenetic reconstruction and continuous spatial diffusion modelling to understand Hungary's role in continental transmission dynamics and inform future preparedness strategies.

## Methods

### WNV surveillance

#### Human surveillance and case definition

Human WNV infections are notifiable in Hungary under Ministerial Decree No. 1998/18 (VI.3.), with surveillance conducted according to European Union (EU) case definition criteria outlined in Decision (EU) 2018/945/EC [9]. The nationwide surveillance system operates through mandatory reporting by healthcare professionals via the national communicable disease notification system, ensuring comprehensive case capture across all healthcare facilities. The Unit of Communicable Disease and Immunisation at the National Center for Public Health and Pharmacy (NCPHP) in Budapest coordinates national surveillance activities and data synthesis. Data are collected from laboratory-confirmed and probable cases using standardised epidemiological investigation forms, which capture detailed information including demographic characteristics, travel history during the 2–14-day incubation period, clinical manifestations, hospitalisation details and outcomes categorised as full recovery, recovery with sequelae or fatality. These epidemiological data were subsequently used to calculate regional incidence rates per 100,000 inhabitants at the Nomenclature of Territorial Units for Statistics level 3 (NUTS3), corresponding to the county level in Hungary, based on the place of exposure.

Although reporting of WNV cases to the ECDC by Hungary started in 2008, alongside the introduction of EU case definitions, all human cases from the study period (2004–24) with available epidemiological data were laboratory investigated, systematically recorded in the national surveillance database and classified as laboratory-confirmed or probable cases.

#### Avian surveillance

Integrated animal surveillance targeted dead birds reported through veterinary networks, with the systematic testing of birds of prey and corvids as sentinel species. A long-established framework for syndromic and passive avian surveillance is operated at the HUN-REN Veterinary Medical Research Institute and the Department of Microbiology and Infectious Diseases, University of Veterinary Medicine during each WNV transmission season [6,10], from June to October each year. For this study, data were extracted for the period June–October 2024.

Information on suspect clinical and mortality cases, with the history of sudden death, debilitation, and neurological symptoms in songbirds, corvids and birds of prey, were collected through professional links with nature conservation organisations, wildlife rehabilitation facilities, falconers, game managers and zoological collections. Samples were also collected in association with ongoing nature conservation and field research projects. All clinical and pathological sampling procedures were performed by the members of our team. Avian cloacal and pharyngeal swabs (Copan Italia) were fixed and stored in 1x Zymo Shield solution (Zymo Research) and standard veterinary postmortem procedures were used for tissue collection (brain, spleen, liver, kidneys, heart).

#### Environmental surveillance

Environmental surveillance comprised targeted mosquito trapping in areas with confirmed human cases, complete with species identification and pathogen testing. Information on the suspected place of exposure of human WNV cases was shared weekly with veterinary health professionals continuously from the detection of the first human case, to support targeted mosquito trapping from 1 August to 31 October 2024. Furthermore, seven trapping sites were continuously operated in and around Budapest as part of a pilot study, where mosquito collection and WNV surveillance were conducted between 9 May and 8 November 2024.

### Laboratory diagnosis and molecular characterisation of WNV

#### Human samples

Centralised laboratory confirmation of human WNV cases is performed exclusively at the national reference laboratory for viral zoonoses at the NCPHP, ensuring standardised testing protocols and quality assurance. Serological testing was performed using commercially available CE-IVD-marked indirect immunofluorescence assays and ELISA tests targeting WNV-specific IgM, IgG and IgA antibodies in serum and cerebrospinal fluid (CSF) samples.

Case confirmation of human WNV infections requires fulfilment of at least one of four criteria: (i) WNV isolation from blood or CSF; (ii) WNV RNA detection in blood or CSF; (iii) WNV-specific IgM detection in CSF; or (iv) high-titre WNV-specific IgM and IgG in blood with neutralisation confirmation. Additional confirmation was based on demonstrated seroconversion between acute and convalescent sera.

Given the considerable cross-reactivity among orthoflaviviruses, the differential diagnosis of WNV cases requires parallel serological testing for TBEV and USUV, along with parallel PCR testing for USUV. West Nile virus detection in human clinical specimens was performed using the RealStar WNV RT-PCR Kit 2.0 (CE-IVD; Altona Diagnostics). During the 2024 seasonal period, indications for WNV and USUV RT-PCR testing, performed for diagnostic purposes, included at least one of the following: (i) the patient being immunocompromised; (ii) presenting with a severe condition, such as requiring intensive care unit treatment; (iii) suspicion of a secondary orthoflavivirus infection, based on results of the serological tests; (iv) whole blood and urine samples were available. For cases i-iii, RT-PCR testing was conducted on all accessible specimen types obtained from each patient (serum or plasma, whole blood, CSF and urine). Furthermore, in all cases, paired serum samples collected 10–14 days apart were requested for serological testing to confirm or exclude current WNV infection.

#### Avian and mosquito samples

For samples isolated from birds and mosquitoes, WNV detection was carried out with a modified in-house multiplex RT-qPCR assay, adapted from the protocol of Del Amo et al. (2013) [11]. Non-human samples in this study were analysed at the HUN-REN Veterinary Medical Research Institute and at the University of Veterinary Medicine, Budapest. All avian samples examined for WNV during 2024 were included in this study and additional two WNV avian isolates were added from the 2023 season. The complete set of mosquito samples collected by our team during 2024 was processed for this research.

### Genomic sequencing and bioinformatic analysis

#### Human samples

Samples positive for WNV underwent amplicon-based next-generation sequencing using the Illumina NextSeq2000 platform. For genome recovery, we employed the sensitive protocol variant accessible on protocols.io (https://www.protocols.io/view/west-nile-virus-orthoflavivirus-nilense-lineage-2-q26g71q98gwz/v2) [12]. Library preparation targeted the complete WNV genome using overlapping amplicons designed to capture WNV-L2 diversity. Sequencing was performed to achieve minimum 10 x coverage across target regions. Bioinformatic processing included quality assessment with FastQC v0.12.1 and adapter trimming with BBDuk v38.84. Clean reads were mapped to WNV reference genomes with Bowtie2 v2.4.5. Consensus sequences were generated using SAMtools v1.18 with minimum coverage (5x) threshold applied. Suitable genome sequences (> 70%) were retained for phylogenetic analysis, yielding 56 genomes from 38 cases. Of the 38 cases, 30 were from 2024, seven from 2023 and one from 2022.

#### Avian and mosquito samples

West Nile virus-positive samples were processed by amplicon-based Nanopore sequencing according to an established protocol [13]. Libraries were prepared with the Ligation Sequencing Kit (SQK-LSK110) and barcoded using Native Barcoding Kits (EXP-NBD104/114, Oxford Nanopore Technologies). Sequencing was performed on a MinION Mk1B device with an R9.4.1 flow cell. Basecalling and demultiplexing were carried out with Dorado v6.5.7. Reads were processed using the VirDetector pipeline with hybrid assembly (de novo and reference-based). Final variant call and filtering were performed using LoFreq, and consensus genomes were generated with BCFtools.

### Phylogeographic reconstruction and spatial analysis

Comprehensive phylogenetic analysis incorporated a European WNV-L2 dataset of 637 sequences spanning 2004–24, derived from humans, animals and mosquito vectors. Sequences were retrieved from NCBI GenBank with associated geographic and temporal metadata, aligned using MAFFT and manually curated to ensure highest accuracy. Time-scaled phylogenetic analysis employed BEAST v1.10.5 with an uncorrelated relaxed molecular clock model and Skygrid coalescent prior. Markov Chain Monte Carlo (MCMC) analyses ran for 400 million steps with sampling every 10,000 steps, ensuring adequate effective sample sizes (> 200) for all parameters. Phylogeographic reconstruction used both discrete and continuous spatial models implemented in BEAST. Discrete trait analysis employed Bayesian Stochastic Search Variable Selection (BSSVS) to identify statistically supported transmission routes between countries. Continuous phylogeography used the Relaxed Random Walk (RRW) framework to model viral diffusion in geographic space, enabling estimation of migration rates and identification of transmission hubs. EvoLaps 2 (www.evolaps.org) was applied to analyse and illustrate WNV spatial dynamics, allowing detailed phylogeographic reconstruction and graphical representation.

## Results

### Epidemiological overview and temporal patterns

**Figure 1:**
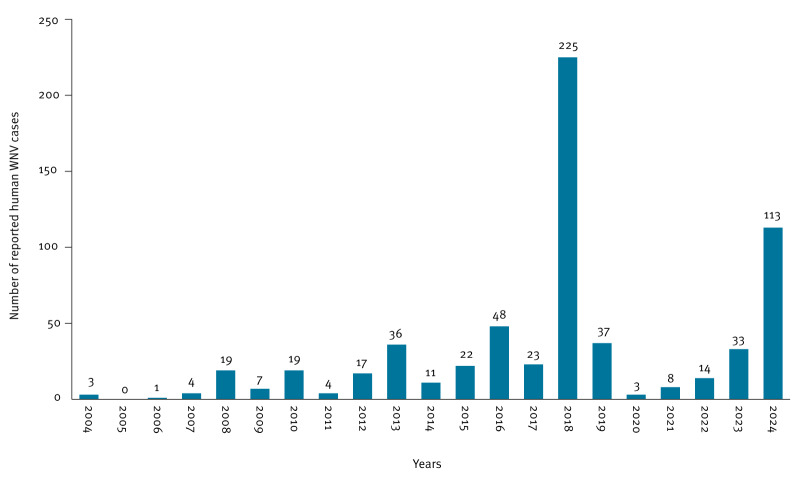
Annual number of reported human WNV cases, Hungary, 2004–2024 (n  =  647)

Human neuroinvasive WNV cases were first documented in Hungary in 2004, and since then, cases have been reported annually, with the largest outbreak occurring in 2018, when 225 cases of WNF and WNND were registered (Figure 1). Hungary reported 113 human WNV cases during the 2024 transmission season, comprising 111 autochthonous and two imported cases. One imported case originated from Greece with documented travel history, while the second case involved multiple-country travel during the incubation period, preventing definitive source attribution. This total represented a substantial 3.7-fold increase compared with 2023, when 30 autochthonous cases were reported (national incidence rates: 1.16 vs 0.31 per 100,000 population) (Figure 1).

WNV: West Nile virus.

The number above each bar represents the total number of reported human West Nile Fever and West Nile neuroinvasive disease cases. Annual case counts comprise both autochthonous and imported cases.

**Figure 2:**
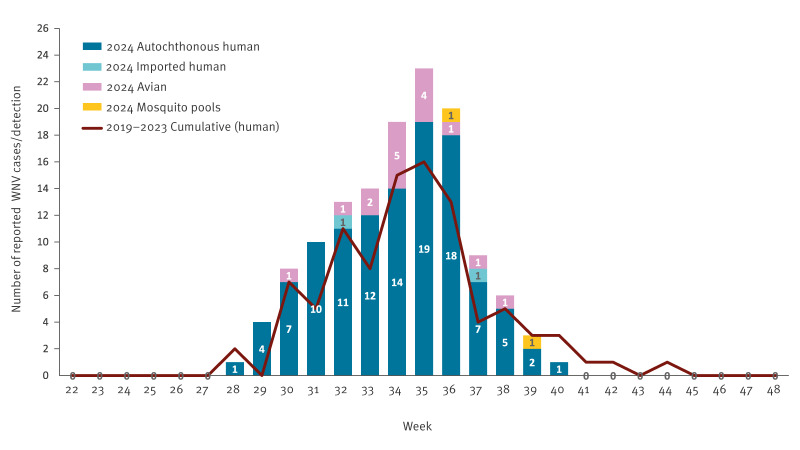
Weekly number of reported human WNV cases (n  =  113), WNV-infected birds (n  =  16) and mosquito pools (n  =  2), Hungary, 2024 compared with cumulative weekly human cases, 2019–2023 (n  =  95)

The 2024 transmission season extended from week 28 to week 40, with the first human case experiencing symptom onset on 12 July and the final case on 2 October. Peak transmission occurred during weeks 35–36 (26 Aug–1 Sep and 2 Sep–8 Sep), accounting for most reported cases (Figure 2). This temporal pattern aligned with historical WNV seasonality in central Europe, although the extended duration until week 40 suggested favourable environmental conditions supporting prolonged vector activity.

WNV: West Nile virus.

Week refers to the date of symptom onset in human cases and to the time of virus detection in birds or in mosquito pools. Reported human cases include West Nile Fever and West Nile neuroinvasive cases. Autochthonous 2024 human cases are shown in dark blue bars, imported human 2024 cases in light bars, 2019–23 cumulative human cases are indicated by the red line. Avian infections are shown in pink bars and infected mosquito pools in orange.

### Clinical and demographic characteristics of human WNV disease

**Figure 3:**
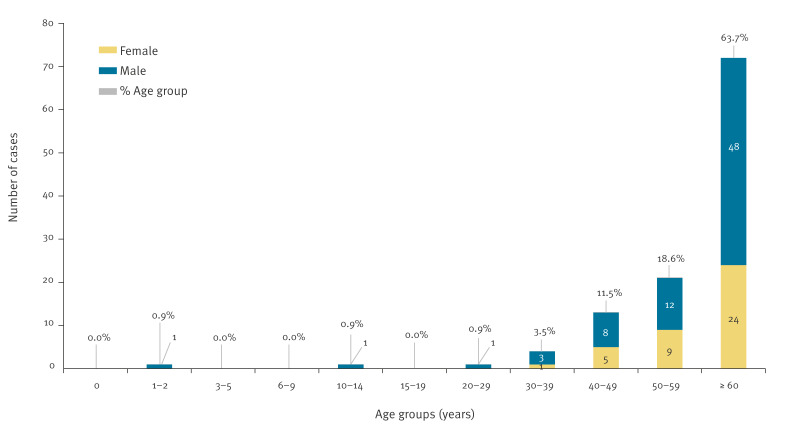
Age and sex distribution of reported human WNV cases, Hungary, 2024 (n  =  113)

Demographic analysis revealed predominance of WNV disease among elderly people, with 63.7% (n = 72) of all 113 cases occurring in individuals aged > 60 years (Figure 3). Age distribution ranged from 2 to 86 years (mean: 63.1 years), consistent with established age-related risk patterns for severe WNV disease. Male cases predominated (65.5%, n = 74), aligning with epidemiological patterns observed across European WNV-endemic regions. Clinical presentation was characterised by high rates of neuroinvasive disease, with 92.0% of patients (n = 104) developing WNND. Hospital admission was required all 104 cases, with 21.2% (n = 22) necessitating intensive care unit treatment at the time of clinical indication for laboratory testing. Case fatality rate (CFR) reached 7.9% (n = 9). Recovery outcomes included full recovery without sequelae in 90.3% (n = 102); recovery with documented neurological or cognitive sequelae occurred in two patients (1.8%). Underlying immunocompromising conditions affected 11.5% (n = 13) of WNND patients, including nine with onco-haematological malignancies, three organ transplant recipients, one with Crohn's disease, and one with systemic lupus erythematosus. One liver transplant recipient presented with diffuse large B-cell lymphoma. This distribution highlighted the vulnerability of immunocompromised populations to severe WNV outcomes.

WNV: West Nile virus.

Bars represent male (dark blue) and female cases (yellow), with the number of cases indicated. Percentages above the bars indicate the proportion of total cases within each age group.

**Figure 4:**
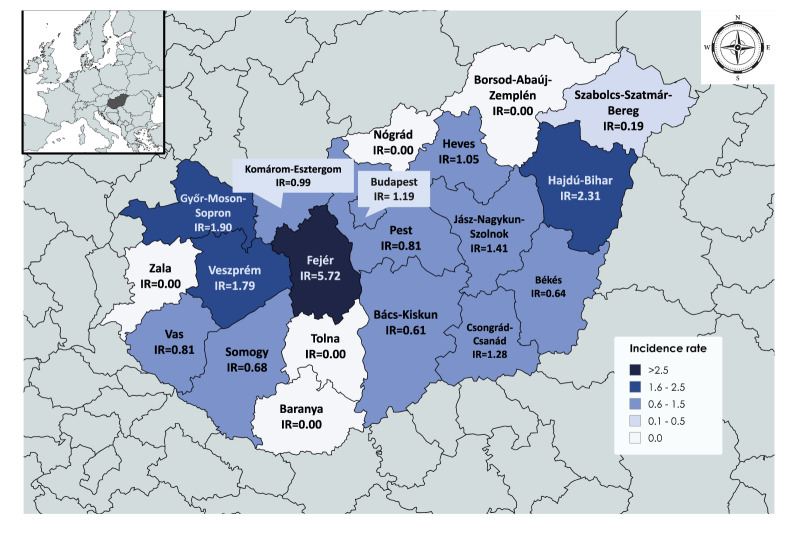
Incidence rates of reported human WNV cases by county, Hungary, 2024 (n  = 108)

West Nile virus circulation was documented across nearly all Hungarian counties at NUTS 3 level, demonstrating widespread geographic distribution. Fejér county in the Central Transdanubian region recorded the highest regional incidence rate (5.72/100,000 population), followed by Hajdú-Bihar (2.31/100,000), Győr-Moson-Sopron (1.90/100,000) and Veszprém (1.79/100,000) (Figure 4). Some counties in the Great Plain region, such as Jász-Nagykun-Szolnok, Csongrád-Csanád and Heves also recorded incidence rates above 1.00 per 100,000 (Figure 4). This geographic pattern reflected the distribution of suitable vector habitat, particularly areas with extensive wetlands, agricultural irrigation systems, and urban water features supporting Culex mosquito populations.

WNV: West Nile virus.

Counties (NUTS 3 level) are shaded according to incidence rate categories (per 100,000 population). The Great Plain region covers the following counties: Hajdú-Bihar, Jász-Nagykun-Szolnok, Szabolcs-Szatmár-Bereg, Bács-Kiskun, Békés, Csongrád-Csanád.

### Microbiological findings and sequencing

#### Human samples

Laboratory confirmation classified 88 human cases as confirmed and 25 as probable, according to the EU case definition criteria. Confirmation was achieved in 51 patients (58%) by detecting WNV-specific IgM in CSF, in three patients (3%) by demonstrating seroconversion, and in 34 patients (39%) by detecting viral RNA in blood or CSF.

RT-qPCR testing was performed on specimens from 77 patients (68% of 113 total cases), yielding positive results in 43 individuals (56% of 77 tested, including additional sample types such as urine). Specimen-specific RT-qPCR detection rates varied considerably: whole blood samples were positive in 31 patients (58% of 53 tested individuals), serum samples in 6 patients (22% of 27), urine samples in 34 patients (58% of 59), and CSF in only seven patients (30% of 23). Among 47 patients with whole blood and urine samples collected at the same time, dual positivity was observed in 24 cases (51% of 47), reflecting the complex viraemic patterns and specimen-dependent detection sensitivity. Interpretation of the PCR results was limited by the inconsistent availability of sample types per patient.

#### Avian and mosquito samples

In total, 132 avian carcasses and 61 pharyngeal and cloacal swab samples from clinically healthy birds were collected from July to mid-October 2024. West Nile virus was detected in organ samples of 15 dead birds and a single swab sample of a clinically healthy bird, and complete WNV genomes were successfully sequenced from 13 dead birds, namely six goshawks (*Accipiter gentilis*), five hooded crows (*Corvus cornix*), one carrion crow (*Corvus corone*), and one greenfinch (*Chloris chloris*). Pharyngeal and cloacal swabs tested WNV-positive and the pharyngeal isolate was sequenced from a clinically healthy little owl (*Athene noctua*). Genomes from two 2023 infections, a clinically ill and euthanised mallard (Anas platyrhynchos) and an African penguin (*Spheniscus demersus*) mortality case, were also included in the phylogenetic analysis.

The targeted mosquito trapping at 31 human outbreak locations from July to September yielded a total of 6,711 female Culex pipiens mosquitoes tested in 422 pools. We detected two infected mosquito pools from which we sequenced complete WNV-L2 genomes confirming local virus circulation. The bias corrected maximum likelihood estimate of a single mosquito infection rate was 0.030 (95% CI: 0.002–0.235) (sampled on 2 Sep 2024) and 0.028 (95% CI: 0.002–0.211) (sampled on 27 Sep 2024) per 1,000 individuals. Pooled infection rates were estimated using PooledInfRate v4.0 (Centers for Disease Control and Prevention, United States) [14].

### Sequencing

Altogether, from the WNV-positive samples, we generated 55 unique novel WNV genomes (human: n  =  38 separate cases, non-human: n  =  17) providing high-resolution genetic data for phylogeographic analysis. The metadata of sequenced samples are available in Supplementary Table S1.

### Phylogenetic diversity of Hungarian WNV strains in the European context

**Figure 5:**
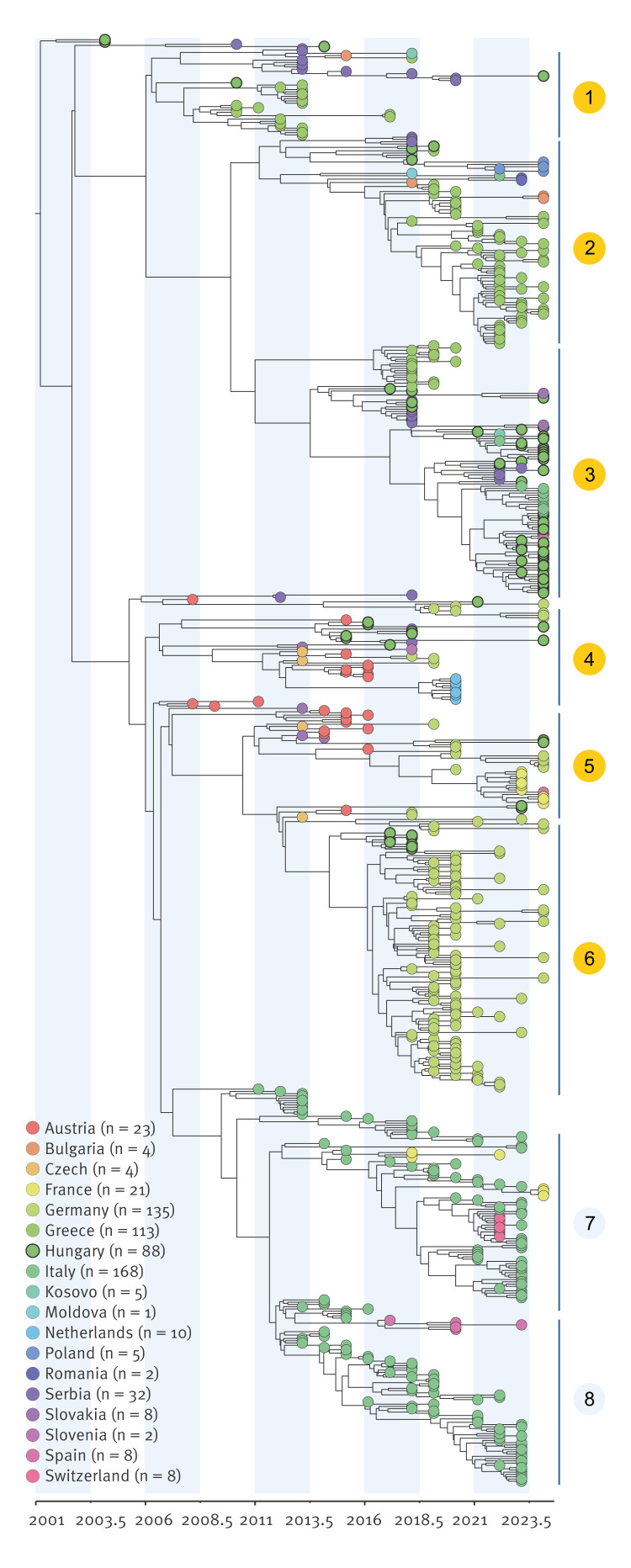
Bayesian maximum clade credibility tree of the WNV lineage 2 Hungary, 2004–2024 (n  =  88 sequences) and Europe, 2004–2024, (n  =  549 sequences)

Phylogenetic analysis of the novel 55 Hungarian sequences generated in this study with European WNV-L2 sequences (n  =  637; 2004–24) revealed Hungarian strains distributed across six of eight major European clades, representing the highest genetic diversity of any single European country (Figure 5, Supplementary Figure S1). The Hungarian genomes clustered within previously established European clades, confirming the continued circulation of locally adapted variants. Time-scaled Bayesian analysis placed the earliest establishment of WNV-L2 in Hungary around 2002–03 (95% highest posterior density (HPD): 2001–05), with subsequent diversification arising through multiple independent introduction events rather than expansion from a single founder (Figure 5). The 2024 genomes showed > 99% nucleotide identity with previously detected Hungarian strains, indicating sustained local transmission, while also clustering with contemporaneous European sequences, reflecting ongoing regional connectivity.

WNV: West Nile virus.

Tip label colours indicate the geographic origin of sampled sequences (see colour key), with clades consisting Hungarian strains (1–6, orange circles). The X-axis shows calendar years relative to the most recent sampling date (2024).

### Phylogeographic transmission dynamics and migration patterns

**Figure 6:**
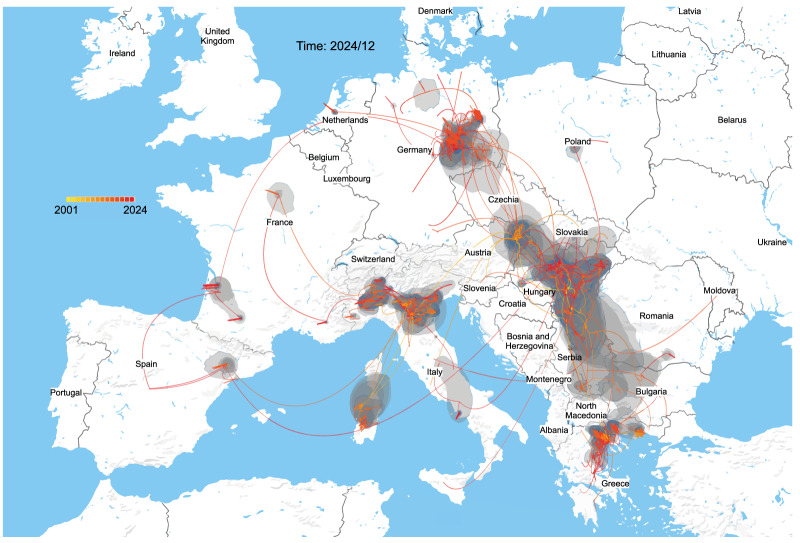
Continuous phylogeographic diffusion of West Nile virus lineage 2 inferred from the Bayesian maximum clade credibility tree, Europe, 2004–2024

Discrete and continuous phylogeographic reconstructions consistently placed Hungary at the basal position of the European WNV-L2 phylogeny, confirming its role as a primary source of regional diversity. Quantitative estimates indicated 18–25 introductions into Hungary over the past 22 years (mean: ca 0.95 events/year) and 32–49 exportations (mean: ca 1.8 events/year), yielding a net migration balance of ca 1.7:1 in favour of exportation. Phylogeographic ancestry confirmed that all Austrian (23/23) and Slovak (8/8) sequences, and 98.5% of German sequences (133/135), traced directly or indirectly to Hungarian variants. Hungary was also a major source of WNV viral populations beyond central Europe. In the Balkans, 94–100% of Serbian sequences and all Greek sequences (113/113) were of Hungarian ancestry; notably, all Polish sequences (5/5) also traced back to Hungary. Continuous diffusion reconstructions additionally suggested occasional long-range dispersal into Italy and Spain (Figure 6). In the discrete BSSVS analysis, 8.3% of Italian sequences (14/168) and 25% of Spanish sequences (2/8) reflected direct introductions from Hungary, while 100% of Italian and Spanish variants showed Hungarian ancestry upstream via intermediaries (Austria, Serbia or Greece). Although the direct Hungary to Austria transition had only modest posterior probability (ca 0.27), all Austrian strains ultimately traced back to Hungarian variants. This reflects that many introductions are reconstructed as two-step events (Hungary–Slovakia–Austria) rather than single direct transitions. Austria, therefore, acted less as an independent source than as a downstream recipient and conduit, with its role becoming particularly evident after 2015, when it served as a stepping stone for onward transmission into Germany (Austria–Germany posterior probability = 1.0).

WNV: West Nile virus.

Lines represent inferred viral dispersal pathways derived from continuous phylogeographic analysis (BEAST RRW model), with directionality indicating spread over time. Line density reflects the frequency of inferred movements. This figure corresponds to the static summary of the spatial diffusion analysis; an animated version is provided as an accompanying MP4 file (Supplementary Film S1).

The combined reconstructions revealed two dominant dispersal corridors. From western Hungary (Transdanubia), WNV variants spread into Austria and further to Germany, Czechia and Poland. From south-eastern Hungary (Pannonian region), dispersal was directed into the Balkans, seeding Serbia and extending to Greece. The phylogeographic reconstruction is provided in Supplementary Figure S2. These parallel routes emphasise Hungary’s dual role as a source for westward expansion into Central Europe and a reservoir for Balkan transmission networks. Within-country analysis showed moderate spatial autocorrelation between genetic and geographic distances (Mantel test Rca 0.34, p < 0.01), consistent with the development of regional transmission foci (Supplementary Figures S1 and S2). Dispersal was strongly distance-dependent: 65% of migration events occurred at short range (< 200 km), primarily to Austria, Slovakia, and Serbia; 28% at medium range (200–500 km), to Czechia and Germany; and 7% at long range (> 500 km), reaching northern Germany, Italy and Spain. Estimated migration rates varied by corridor: 45–67 km/year along the Danube into Germany, and 38–52 km/year through Carpathian Basin networks into Slovakia and Czechia. The 2024 genomes, therefore, confirm the continuity of established Hungarian strains rather than the emergence of novel variants, reflecting stable local transmission. However, the consistently high number of exportations highlights Hungary’s role as both a reservoir and amplifier of WNV diversity across Central, Balkan and Western Europe.

## Discussion

The 2024 WNV transmission season in Hungary, characterised by 113 human cases representing a 3.7-fold increase from 2023, occurred within Europe's most geographically extensive WNV outbreak on record [5]. With 1,436 locally acquired cases across 19 countries and transmission documented in 212 regions, 2024 represented the largest geographic distribution in European surveillance history. Hungary's case numbers ranked fourth highest after Italy (n  =  455), Greece (n  =  217), and Spain (n  =  138), positioning the country among the most severely affected European countries. The extended Hungarian transmission season from weeks 28–40 aligns with unprecedented European climate conditions [15]. The convergence of record-breaking spring temperatures ( + 1.50 °C above average), the hottest European summer on record ( + 1.54 °C), and extreme drought conditions in south-eastern Europe provided ideal conditions for virus circulation. These climate extremes, combined with September flooding from Storm Boris, sustained transmission well beyond historical norms.

Recent climate attribution studies demonstrate that climate change has been a critical driver of WNV spatial expansion in Europe [16], with the 2024 season exemplifying how environmental extremes can amplify disease transmission. The 2024 outbreak shares climatic parallels with the 2018 European epidemic, which was similarly preceded by favourable environmental conditions for WNV circulation including increased temperatures and a wet spring followed by drought [17]. While the 2018 season recorded higher absolute case numbers (2,083 vs 1,436), the 2024 season exhibited the largest geographical distribution in European surveillance history, affecting 19 countries, indicating continued expansion of transmission-suitable areas under climate change.

Our phylogeographic findings, which establish Hungary as the primary source of European WNV diversity with Hungarian strains distributed across six of eight major European L2 clades, align with extensive recent molecular epidemiology research [18,19]. While Hungary has previously been recognised as a transmission hub, our study provides, to our knowledge, the first quantitative, genome-resolved evidence demonstrating that the country harbours among the highest WNV-L2 genetic diversity observed in Europe and serves as a net exporter of viral diversity. Multiple independent phylogeographic studies from 2017 to 2024 consistently identify Hungary alongside Austria as the primary ‘radiation centres’ for European WNV-L2 circulation [20,21]. The most recent comprehensive European analysis by Lu et al. (2024) documented that European WNV-2a evolved into two major co-circulating clusters, with cluster B having its ancestral origin in Hungary around June 2007 and subsequently spreading predominantly to Greece, Romania and Bulgaria [18]. The observed net migration balance of 1.7:1 favouring viral exportation from Hungary is supported by discrete phylogeographic analyses, identifying Hungary as predominantly acting as a source rather than sink in European transmission networks [20,22].

While Hungary also receives viral introductions, our phylogeographic results demonstrate that it currently functions as a major net exporter of WNV lineages in Europe. This does not exclude local amplification in neighbouring countries but highlights Hungary's central role within the European transmission network. This persistent role as an ecological niche for viral maintenance and export has been attributed to Hungary's position at the intersection of major African–European bird migration flyways and its extensive agricultural landscapes supporting both competent vector populations and amplifying bird hosts [23]. The two dominant dispersal corridors identified, westward via the Danube to Central Europe and south-eastward to the Balkans correspond directly to phylogeographically documented transmission routes [18,19]. The Danube corridor facilitates viral movement through interconnected wetland systems supporting both migratory bird stopovers and dense Culex pipiens populations, while the Balkan route follows established patterns of viral dissemination through the Hungary–Serbia–Bulgaria–Greece pathway [21,24].

The observed rate of 92% neuroinvasive disease development among confirmed Hungarian cases markedly exceeds typical European surveillance data, where WNND proportions generally range from 70 to 80% of symptomatic cases [25]. This discrepancy likely reflects surveillance bias towards severe cases requiring medical attention rather than intrinsic differences in virulence. The 2018 European epidemic documented 70% WNND among symptomatic cases, while earlier years showed rates of 82% (2017) and 86% (2016) [5]. The CFR of 7.9% observed in Hungary falls within the documented European range but represents favourable clinical outcomes compared with several European countries [25]. Recent systematic analyses of European WNV outcomes document CFRs ranging from 3.0% to > 20.0% for WNND cases, with countries like Serbia reporting 17.3% mortality among WNND patients [25,26]. The relatively lower Hungarian CFR may reflect effective clinical management protocols, early diagnosis or demographic differences in affected populations. European seroprevalence studies suggest that symptomatic infections represent only 20–25% of total infections, with neuroinvasive disease occurring in ca 1% of all WNV infections [27].

The 2024 Hungarian outbreak highlights critical priorities for enhanced European WNV surveillance and preparedness. Implementation of truly integrated One Health surveillance systems should be prioritised, following successful models developed in northern Italy, where multi-sectoral coordination enabled virus detection nine days before human symptom onset [28]. Climate adaptation strategies must be incorporated into surveillance planning as projected fivefold increases in WNV risk across Europe by 2040–60 will expand the geographic scope of transmission and extend seasonal activity [29]. Development of climate-informed early warning systems could enable proactive public health responses and targeted vector control interventions [30]. Phylogeographic surveillance should be expanded to include systematic whole genome sequencing of human, animal, and vector isolates to better understand real-time transmission dynamics and source attribution [18]. Notably, preliminary data from the 2025 transmission season indicate substantially reduced WNV activity across Europe compared with 2024, with Hungary reporting only 14 autochthonous human cases by 10 December 2025 [31]. This inter-annual variability, likely influenced by immunity levels in bird populations and ecological conditions, underscores the unpredictable nature of WNV epidemiology and the need for sustained surveillance efforts.

Our study had some limitations. Firstly, the phylogeographic analysis faces constraints common to molecular epidemiology studies [32], including sampling bias towards clinically apparent human cases that may not fully represent viral diversity in enzootic cycles, and temporal gaps in sequence availability across countries that can affect reconstruction of transmission dynamics. Secondly, the high proportion of neuroinvasive disease (92%) likely reflects surveillance bias towards severe cases requiring hospitalisation rather than intrinsic virulence differences. Finally, the interpretation of PCR detection rates was constrained by inconsistent sample type availability per patient. Due to the absence of systematic blood donor screening during the study period, genomic data from asymptomatic infections were not available, potentially underrepresenting the full spectrum of viral diversity. In addition, no equine samples were included, limiting insights into transmission dynamics in an important sentinel host.

## Conclusions

The 2024 WNV outbreak in Hungary reflects broader European epidemiological trends and confirms the country’s central role in continental viral ecology. Record-breaking climate conditions, Hungary's position as a primary source of European viral diversity, and its location at key ecological interfaces created conditions for one of Europe’s most notable transmission seasons. These findings highlight the need for further strengthened One Health surveillance, climate-adapted preparedness, and coordinated European responses to an expanding and intensifying public health threat.

## Data Availability

The genomic sequences obtained in this study are available in the GenBank under accession numbers (PV596716-PV605553, PV637092-PV637093, PV693346-PV693361), and the corresponding raw sequencing data were deposited in the Sequence Read Archive (SRA) under Bioproject ID: PRJNA1327097. Corresponding SRA, Biosample and GenBank accession numbers are available in Supplementary Table S1.

## Supplementary Data

3756000 SupplementaryFigures

## Supplementary Data

118000 SupplementaryTable

## Supplementary Data

25746000 SupplementaryFilm
