# Supplementary material for: Hungary as a source of West Nile virus diversity and spread in Europe: insights from the 2024 transmission season
Source: Euro Surveill. 2026 Apr 23;31(16):2500785. doi: 10.2807/1560-7917.ES.2026.31.16.2500785 (PMC13109698; doi:10.2807/1560-7917.ES.2026.31.16.2500785)
Supplement: SupplementaryFigures [file 25-00785_NAGY_Supplementary_Figures.pdf]

Supplementary Figure S1. **A**. Geographic distribution of WNV lineage 2 clades in Hungary. Coloured circles represent WNV cases assigned to clades 1-6 based on phylogenetic analysis, with numbers inside circles indicating multiple cases detected at the same or very close location. **B**. Geographic distribution of WNV detections in Hungary, 2024, by host species. Coloured circles represent cases identified in humans (red), birds (blue), horses (light green), mosquitoes (orange), and penguin (green). Numbers inside circles indicate multiple detections at the same or very close location.

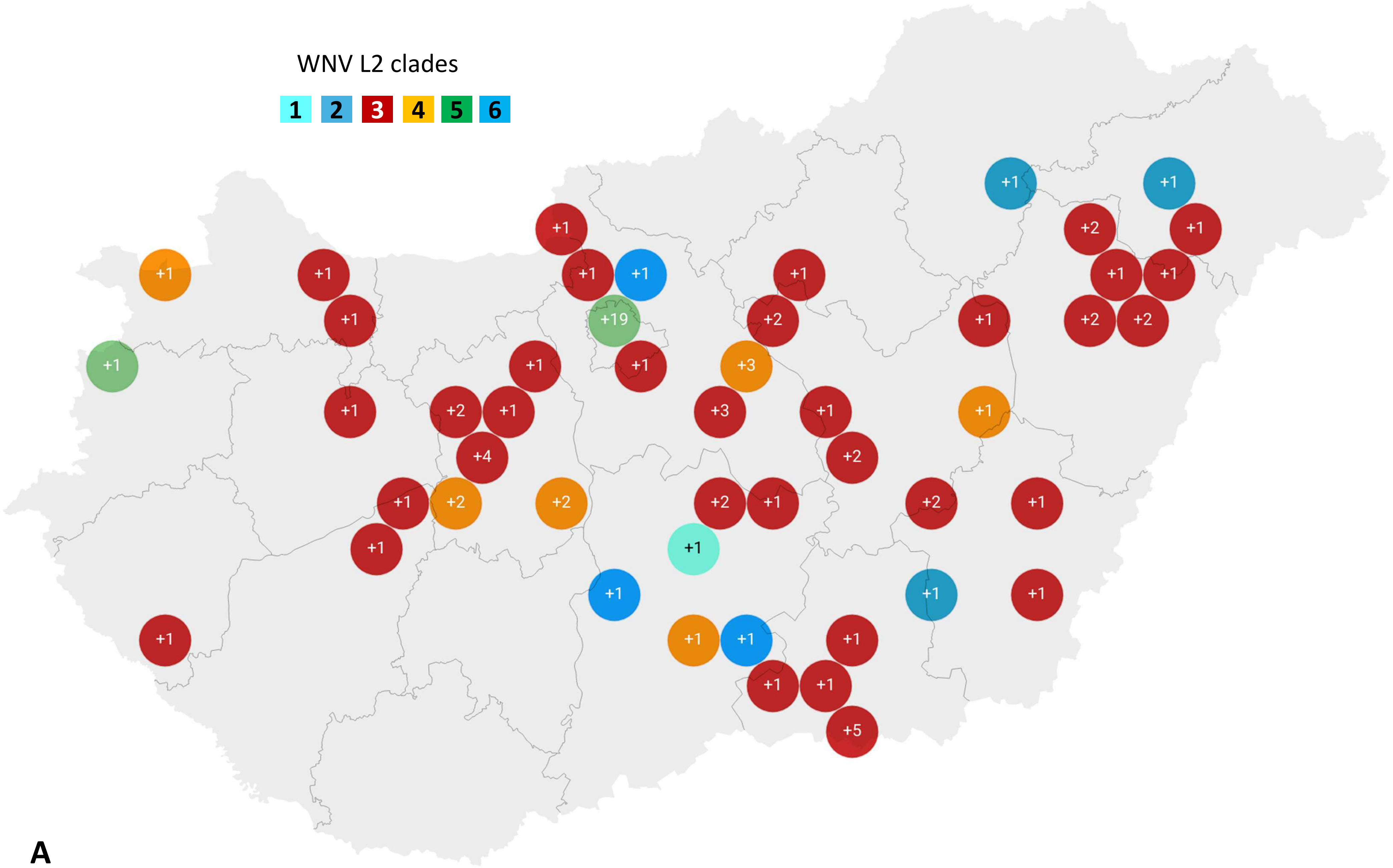

A

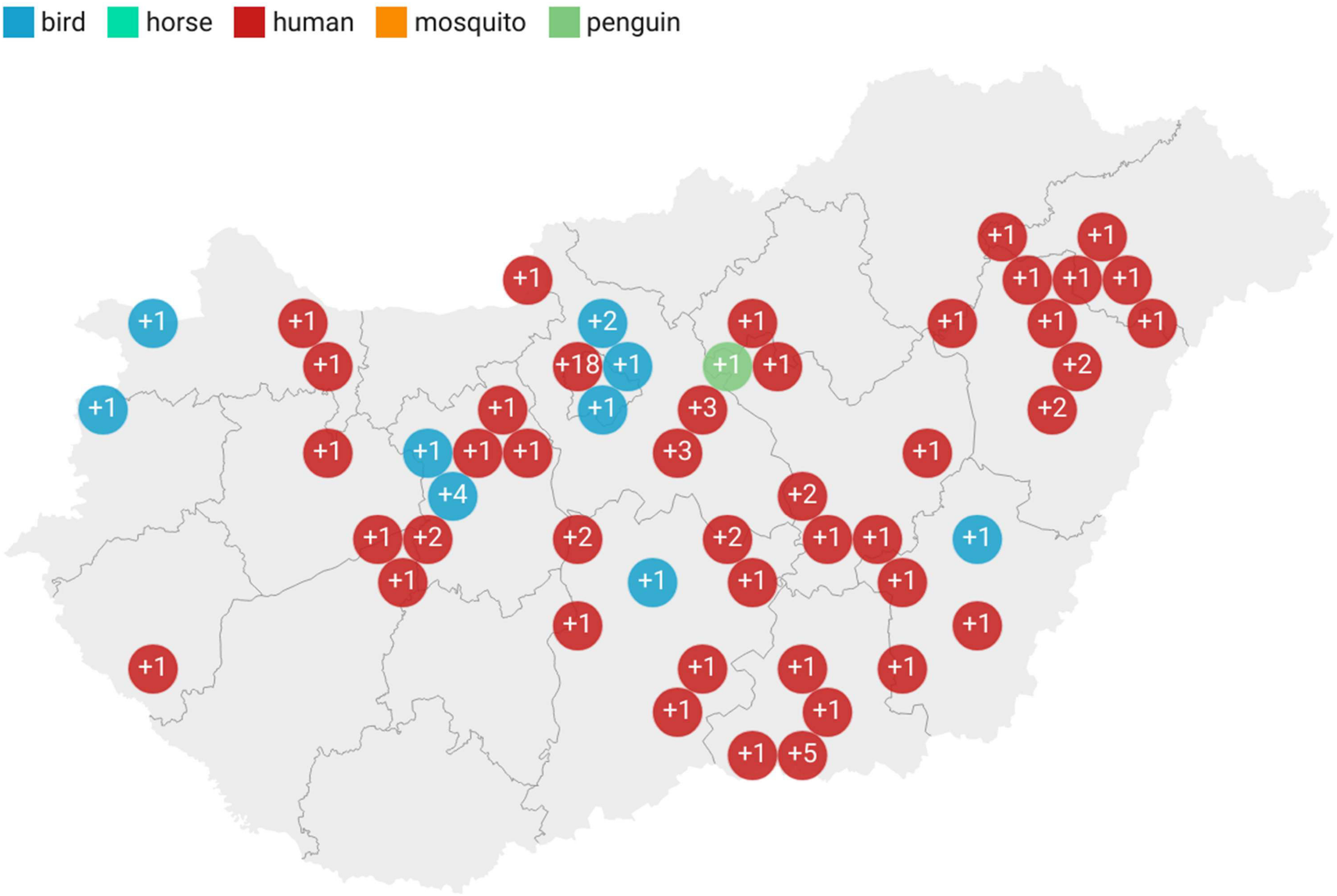

B

Supplementary Figure S2. Phylogeographic reconstruction of individual clades from the European WNV lineage 2a phylogeny (2004–2024) including Hungarian variants. Each panel corresponds to one major clade highlighted in the overall European WNV L2 phylogeny and presents: Discrete analysis (left): A Bayesian maximum clade credibility (MCC) tree showing the time-scaled phylogeny of the selected clade, based on complete or near-complete (≥70%) genome sequences. Branch colours indicate the most probable geographic origin of descendant nodes (see colour key). Hungarian variants, and clade designations correspond to their placement within the broader European tree are highlighted. The x-axis represents calendar years before the most recent sampling date (2024); Continuous analysis (bottom-right panel): The corresponding spatial diffusion map generated in EvoLaps. Viral dispersal is shown as branches representing inferred migration pathways, overlaid with 80% highest posterior density (HPD) regions that reflect phylogeographic uncertainty. Branch colours indicate time, ranging from yellow (time to the most recent common ancestor, TMRCA) to red (most recent sampling time). Grey shaded HPD regions highlight zones of concentrated viral activity and transmission hubs.

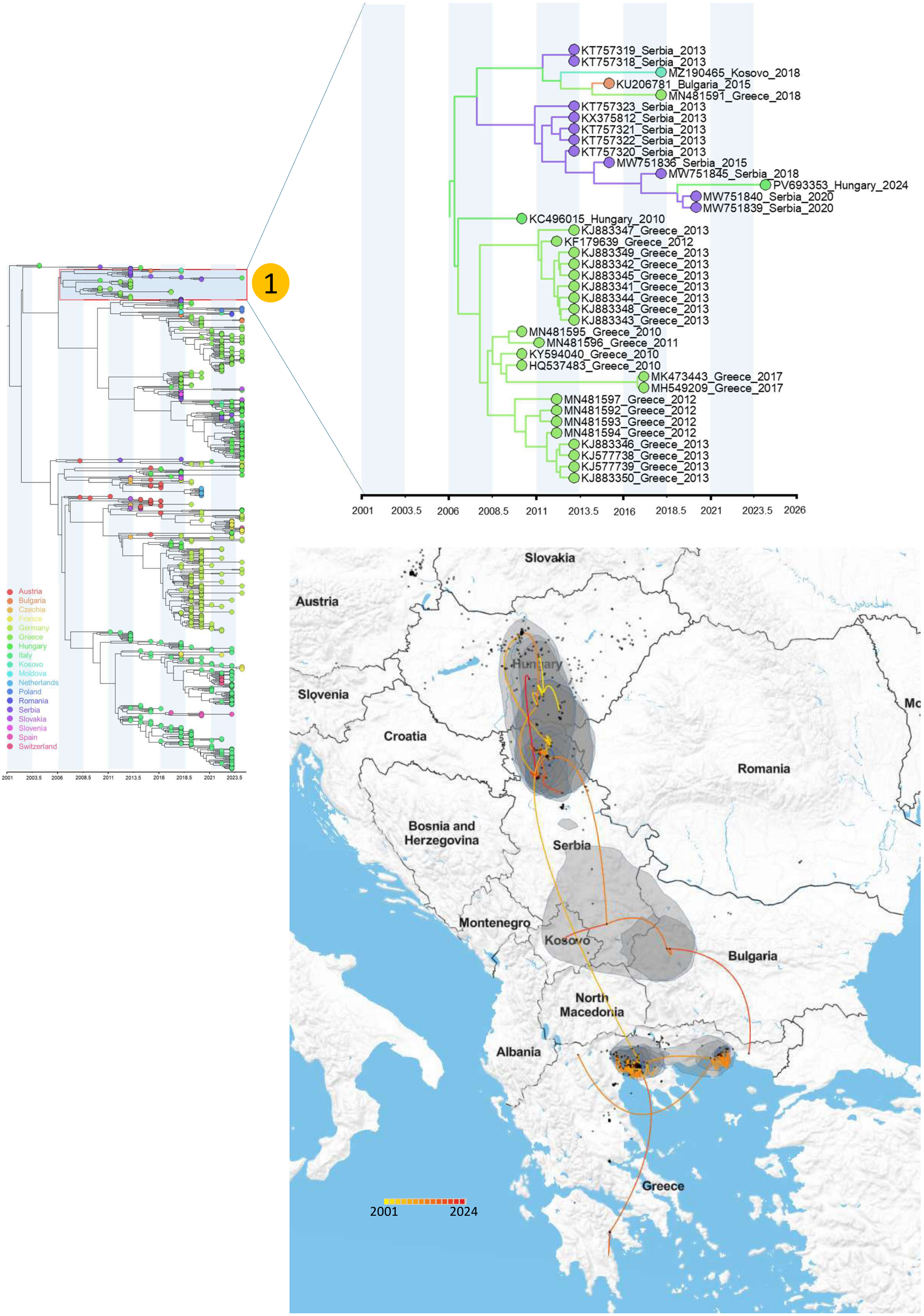
Austria
Bulgaria
Czechia
France
Germany
Greece
Hungary
Italy
Kosovo
Moldova
Netherlands
Poland
Romania
Serbia
Slovakia
Slovenia
Spain
Switzerland
2001
2003.5
2006
2008.5
2011
2013.5
2016
2018.5
2021
2023.5
1
KT757319_Serbia_2013
KT757318_Serbia_2013
MZ190465_Kosovo_2018
KU206781_Bulgaria_2015
MN481591_Greece_2018
KT757323_Serbia_2013
KX375812_Serbia_2013
KT757321_Serbia_2013
KT757322_Serbia_2013
KT757320_Serbia_2013
MW751836_Serbia_2015
MW751845_Serbia_2018
PV693353_Hungary_2024
MW751840_Serbia_2020
MW751839_Serbia_2020
KC496015_Hungary_2010
KJ883347_Greece_2013
KF179639_Greece_2012
KJ883349_Greece_2013
KJ883342_Greece_2013
KJ883345_Greece_2013
KJ883341_Greece_2013
KJ883344_Greece_2013
KJ883348_Greece_2013
KJ883343_Greece_2013
MN481595_Greece_2010
MN481596_Greece_2011
KY594040_Greece_2010
HQ537483_Greece_2010
MK473443_Greece_2017
MH549209_Greece_2017
MN481597_Greece_2012
MN481592_Greece_2012
MN481593_Greece_2012
MN481594_Greece_2012
KJ883346_Greece_2013
KJ577738_Greece_2013
KJ577739_Greece_2013
KJ883350_Greece_2013
2001
2003.5
2006
2008.5
2011
2013.5
2016
2018.5
2021
2023.5
2026
Slovakia
Austria
Hungary
Slovenia
Croatia
Romania
Bosnia and Herzegovina
Serbia
Montenegro
Kosovo
Bulgaria
North Macedonia
Albania
Greece
2001
2024

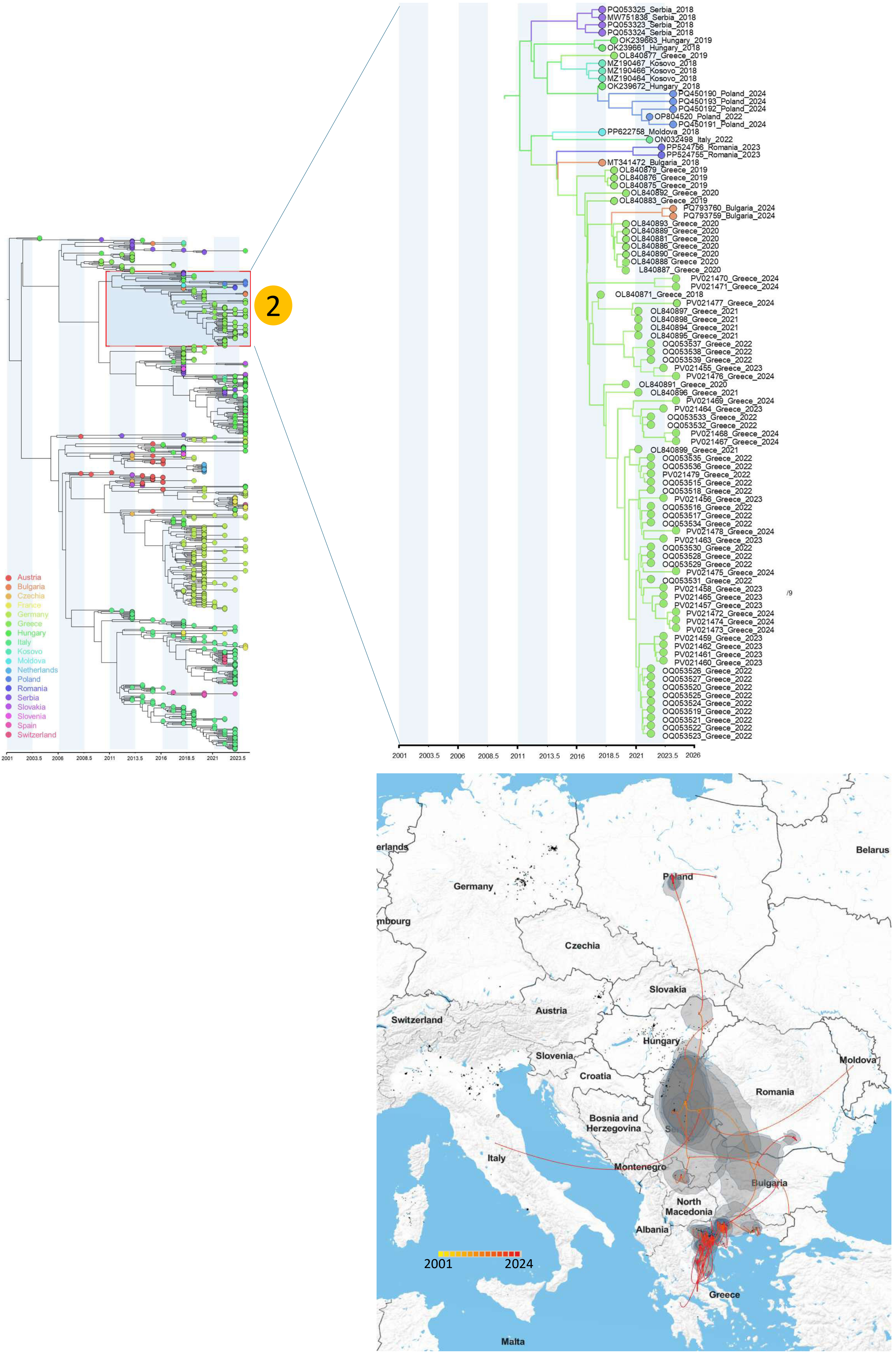

2
Austria
Bulgaria
Czechia
France
Germany
Greece
Hungary
Italy
Kosovo
Moldova
Netherlands
Poland
Romania
Serbia
Slovakia
Slovenia
Spain
Switzerland
2001
2003.5
2006
2008.5
2011
2013.5
2016
2018.5
2021
2023.5
PQ053325_Serbia_2018
MW751838_Serbia_2018
PQ053323_Serbia_2018
PQ053324_Serbia_2018
OK239663_Hungary_2019
OK239661_Hungary_2018
OL840877_Greece_2019
MZ190467_Kosovo_2018
MZ190466_Kosovo_2018
MZ190464_Kosovo_2018
OK239672_Hungary_2018
PQ450190_Poland_2024
PQ450193_Poland_2024
PQ450192_Poland_2024
OP804520_Poland_2022
PQ450191_Poland_2024
PP622758_Moldova_2018
ON032498_Italy_2022
PP524756_Romania_2023
PP524755_Romania_2023
MT341472_Bulgaria_2018
OL840879_Greece_2019
OL840876_Greece_2019
OL840875_Greece_2019
OL840892_Greece_2020
OL840883_Greece_2019
PQ793760_Bulgaria_2024
PQ793759_Bulgaria_2024
OL840893_Greece_2020
OL840889_Greece_2020
OL840881_Greece_2020
OL840886_Greece_2020
OL840890_Greece_2020
OL840888_Greece_2020
L840887_Greece_2020
PV021470_Greece_2024
PV021471_Greece_2024
OL840871_Greece_2018
PV021477_Greece_2024
OL840897_Greece_2021
OL840898_Greece_2021
OL840894_Greece_2021
OL840895_Greece_2021
OQ053537_Greece_2022
OQ053538_Greece_2022
OQ053539_Greece_2022
PV021455_Greece_2023
PV021476_Greece_2024
OL840891_Greece_2020
OL840896_Greece_2021
PV021469_Greece_2024
PV021464_Greece_2023
OQ053533_Greece_2022
OQ053532_Greece_2022
PV021468_Greece_2024
PV021467_Greece_2024
OL840899_Greece_2021
OQ053535_Greece_2022
OQ053536_Greece_2022
PV021479_Greece_2022
OQ053515_Greece_2022
OQ053518_Greece_2022
PV021456_Greece_2023
OQ053516_Greece_2022
OQ053517_Greece_2022
OQ053534_Greece_2022
PV021478_Greece_2024
PV021463_Greece_2023
OQ053530_Greece_2022
OQ053528_Greece_2022
OQ053529_Greece_2022
PV021475_Greece_2024
OQ053531_Greece_2022
PV021458_Greece_2023
PV021465_Greece_2023
PV021457_Greece_2023
PV021472_Greece_2024
PV021474_Greece_2024
PV021473_Greece_2024
PV021459_Greece_2023
PV021462_Greece_2023
PV021461_Greece_2023
PV021460_Greece_2023
OQ053526_Greece_2022
OQ053527_Greece_2022
OQ053520_Greece_2022
OQ053525_Greece_2022
OQ053524_Greece_2022
OQ053519_Greece_2022
OQ053521_Greece_2022
OQ053522_Greece_2022
OQ053523_Greece_2022
/9
2001
2003.5
2006
2008.5
2011
2013.5
2016
2018.5
2021
2023.5
2026
erlands
Belarus
Poland
Germany
mbourg
Czechia
Slovakia
Austria
Switzerland
Hungary
Slovenia
Moldova
Croatia
Romania
Bosnia and Herzegovina
Italy
Montenegro
Bulgaria
North Macedonia
Albania
2001
2024
Greece
Malta

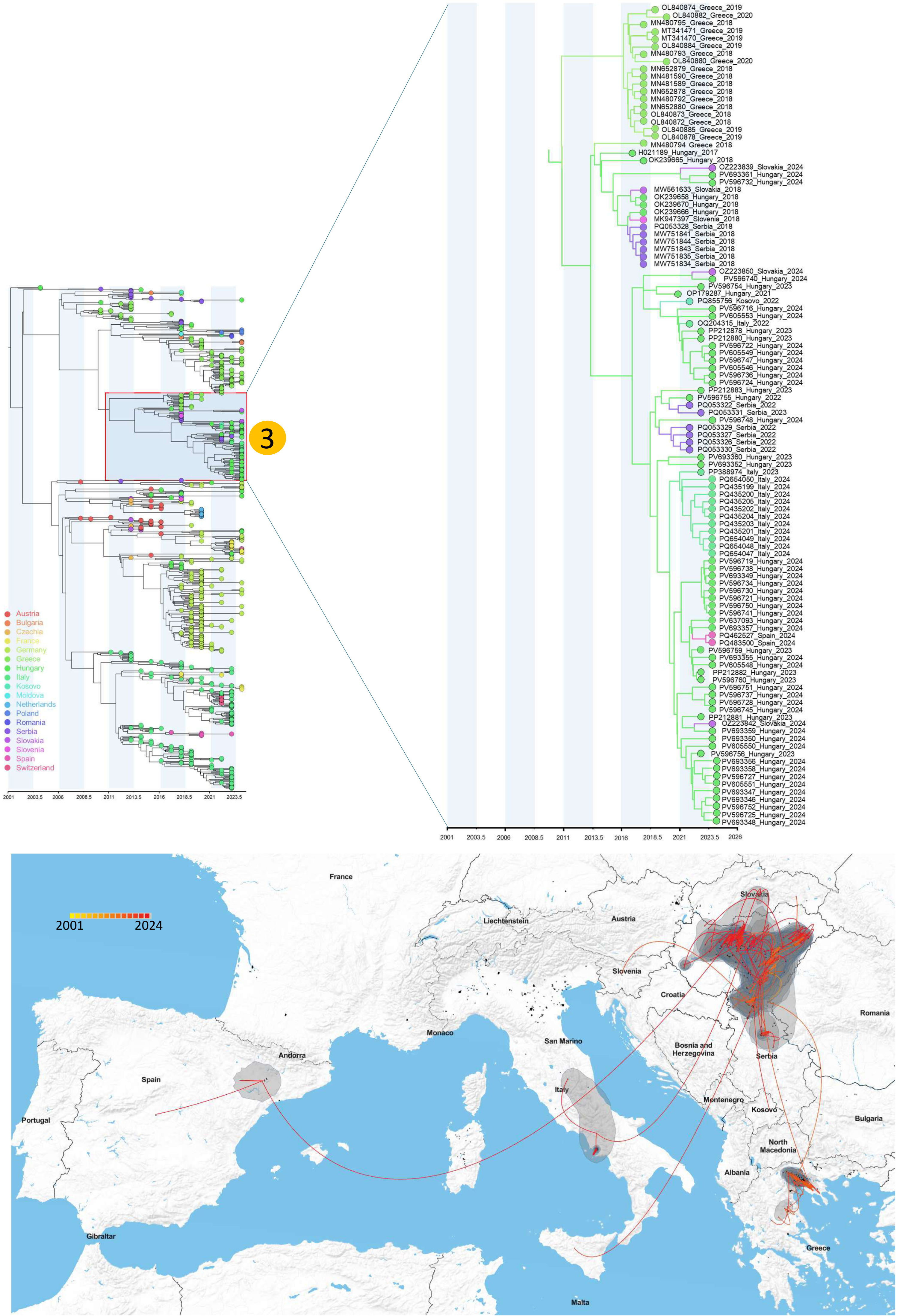

Austria
Bulgaria
Czechia
France
Germany
Greece
Hungary
Italy
Kosovo
Moldova
Netherlands
Poland
Romania
Serbia
Slovakia
Slovenia
Spain
Switzerland
2001
2003.5
2006
2008.5
2011
2013.5
2016
2018.5
2021
2023.5
2026
3
OL840874_Greece_2019
OL840882_Greece_2020
MN480795_Greece_2018
MT341471_Greece_2019
MT341470_Greece_2019
OL840884_Greece_2019
MN480793_Greece_2018
OL840880_Greece_2020
MN652879_Greece_2018
MN481590_Greece_2018
MN481589_Greece_2018
MN652878_Greece_2018
MN480792_Greece_2018
MN652880_Greece_2018
OL840873_Greece_2018
OL840872_Greece_2018
OL840885_Greece_2019
OL840878_Greece_2019
MN480794_Greece_2018
H021189_Hungary_2017
OK239665_Hungary_2018
OZ223839_Slovakia_2024
PV693361_Hungary_2024
PV596732_Hungary_2024
MW561633_Slovakia_2018
OK239658_Hungary_2018
OK239670_Hungary_2018
OK239666_Hungary_2018
MK947397_Slovenia_2018
PQ053328_Serbia_2018
MW751841_Serbia_2018
MW751844_Serbia_2018
MW751843_Serbia_2018
MW751835_Serbia_2018
MW751834_Serbia_2018
OZ223850_Slovakia_2024
PV596740_Hungary_2024
PV596754_Hungary_2023
OP179287_Hungary_2021
PQ855756_Kosovo_2022
PV596716_Hungary_2024
PV605553_Hungary_2024
OQ204315_Italy_2022
PP212878_Hungary_2023
PP212880_Hungary_2023
PV596722_Hungary_2024
PV605549_Hungary_2024
PV596747_Hungary_2024
PV605546_Hungary_2024
PV596736_Hungary_2024
PV596724_Hungary_2024
PP212883_Hungary_2023
PV596755_Hungary_2022
PQ053322_Serbia_2022
PQ053331_Serbia_2023
PV596748_Hungary_2024
PQ053329_Serbia_2022
PQ053327_Serbia_2022
PQ053326_Serbia_2022
PQ053330_Serbia_2022
PV693360_Hungary_2023
PV693352_Hungary_2023
PP388974_Italy_2023
PQ654050_Italy_2024
PQ435199_Italy_2024
PQ435200_Italy_2024
PQ435205_Italy_2024
PQ435202_Italy_2024
PQ435204_Italy_2024
PQ435203_Italy_2024
PQ435201_Italy_2024
PQ654049_Italy_2024
PQ654048_Italy_2024
PQ654047_Italy_2024
PV596719_Hungary_2024
PV596738_Hungary_2024
PV693349_Hungary_2024
PV596734_Hungary_2024
PV596730_Hungary_2024
PV596721_Hungary_2024
PV596750_Hungary_2024
PV596741_Hungary_2024
PV637093_Hungary_2024
PV693357_Hungary_2024
PQ462527_Spain_2024
PQ483500_Spain_2024
PV596759_Hungary_2023
PV693355_Hungary_2024
PV605548_Hungary_2024
PP212882_Hungary_2023
PV596760_Hungary_2023
PV596751_Hungary_2024
PV596737_Hungary_2024
PV596728_Hungary_2024
PV596745_Hungary_2024
PP212881_Hungary_2023
OZ223842_Slovakia_2024
PV693359_Hungary_2024
PV693350_Hungary_2024
PV605550_Hungary_2024
PV596756_Hungary_2023
PV693356_Hungary_2024
PV693358_Hungary_2024
PV596727_Hungary_2024
PV605551_Hungary_2024
PV693347_Hungary_2024
PV693346_Hungary_2024
PV596752_Hungary_2024
PV596725_Hungary_2024
PV693348_Hungary_2024
2001
2024
France
Liechtenstein
Austria
Slovakia
Slovenia
Croatia
Romania
Monaco
San Marino
Bosnia and Herzegovina
Serbia
Andorra
Spain
Italy
Montenegro
Kosovo
Bulgaria
Portugal
North Macedonia
Albania
Gibraltar
Greece
Malta

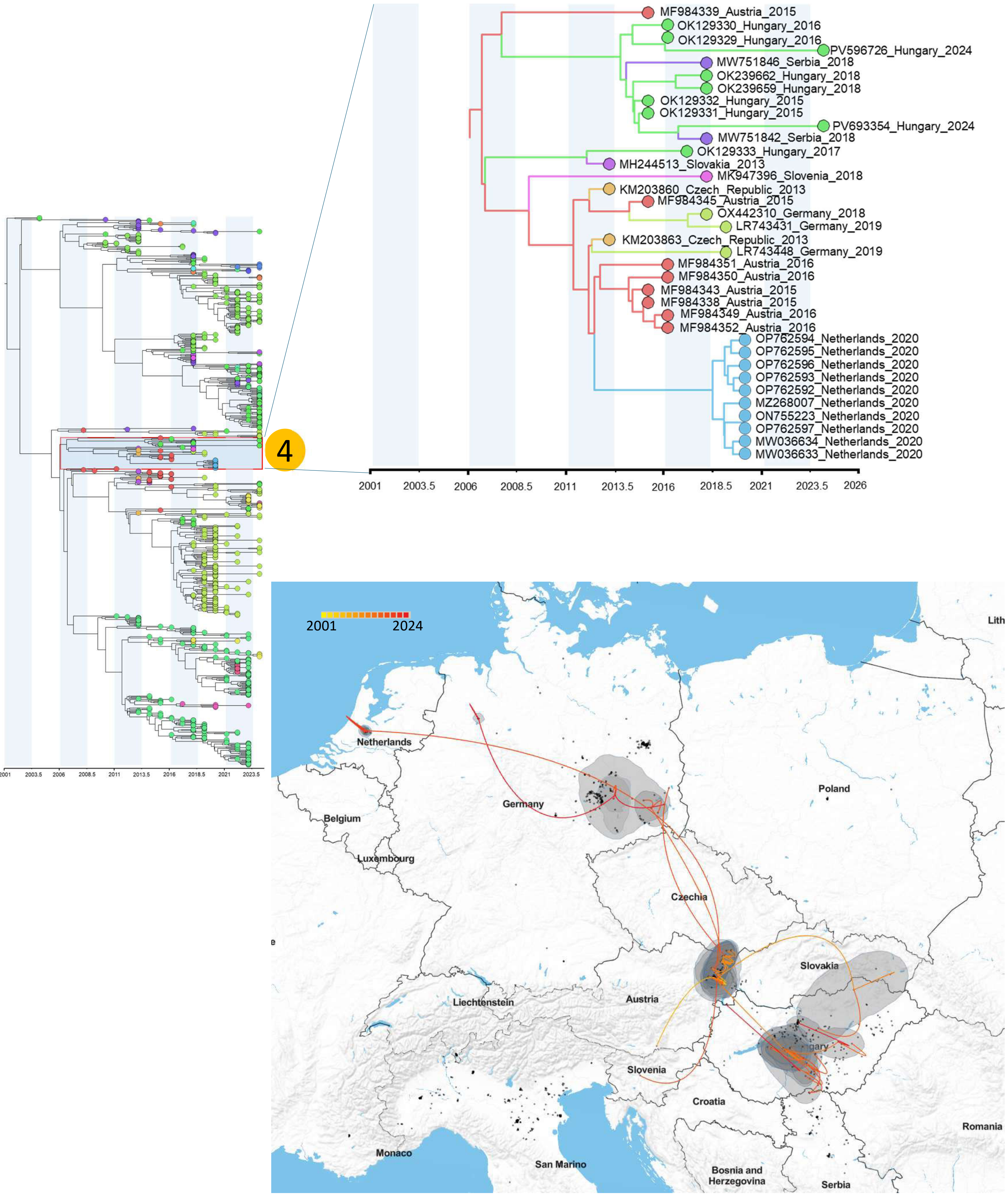
4
MF984339_Austria_2015
OK129330_Hungary_2016
OK129329_Hungary_2016
PV596726_Hungary_2024
MW751846_Serbia_2018
OK239662_Hungary_2018
OK239659_Hungary_2018
OK129332_Hungary_2015
OK129331_Hungary_2015
PV693354_Hungary_2024
MW751842_Serbia_2018
OK129333_Hungary_2017
MH244513_Slovakia_2013
MK947396_Slovenia_2018
KM203860_Czech_Republic_2013
MF984345_Austria_2015
OX442310_Germany_2018
LR743431_Germany_2019
KM203863_Czech_Republic_2013
LR743448_Germany_2019
MF984351_Austria_2016
MF984350_Austria_2016
MF984343_Austria_2015
MF984338_Austria_2015
MF984349_Austria_2016
MF984352_Austria_2016
OP762594_Netherlands_2020
OP762595_Netherlands_2020
OP762596_Netherlands_2020
OP762593_Netherlands_2020
OP762592_Netherlands_2020
MZ268007_Netherlands_2020
ON755223_Netherlands_2020
OP762597_Netherlands_2020
MW036634_Netherlands_2020
MW036633_Netherlands_2020
2001
2003.5
2006
2008.5
2011
2013.5
2016
2018.5
2021
2023.5
2026
2001
2024
Netherlands
Germany
Poland
Belgium
Luxembourg
Czechia
Slovakia
Austria
Liechtenstein
Slovenia
Croatia
Romania
Monaco
San Marino
Bosnia and Herzegovina
Serbia

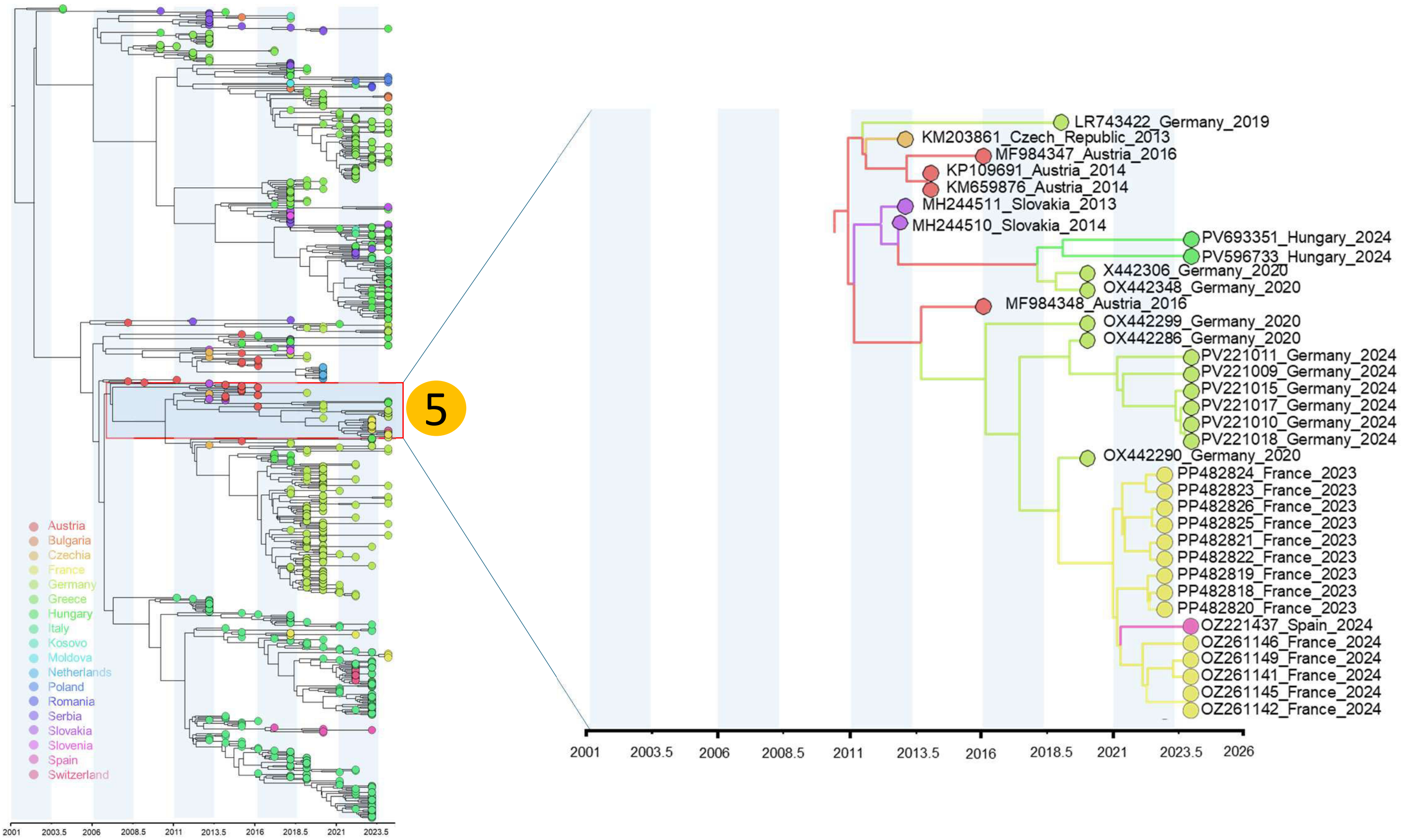
Austria
Bulgaria
Czechia
France
Germany
Greece
Hungary
Italy
Kosovo
Moldova
Netherlands
Poland
Romania
Serbia
Slovakia
Slovenia
Spain
Switzerland
2001
2003.5
2006
2008.5
2011
2013.5
2016
2018.5
2021
2023.5
5
LR743422_Germany_2019
KM203861_Czech_Republic_2013
MF984347_Austria_2016
KP109691_Austria_2014
KM659876_Austria_2014
MH244511_Slovakia_2013
MH244510_Slovakia_2014
PV693351_Hungary_2024
PV596733_Hungary_2024
X442306_Germany_2020
OX442348_Germany_2020
MF984348_Austria_2016
OX442299_Germany_2020
OX442286_Germany_2020
PV221011_Germany_2024
PV221009_Germany_2024
PV221015_Germany_2024
PV221017_Germany_2024
PV221010_Germany_2024
PV221018_Germany_2024
OX442290_Germany_2020
PP482824_France_2023
PP482823_France_2023
PP482826_France_2023
PP482825_France_2023
PP482821_France_2023
PP482822_France_2023
PP482819_France_2023
PP482818_France_2023
PP482820_France_2023
OZ221437_Spain_2024
OZ261146_France_2024
OZ261149_France_2024
OZ261141_France_2024
OZ261145_France_2024
OZ261142_France_2024
2001
2003.5
2006
2008.5
2011
2013.5
2016
2018.5
2021
2023.5
2026

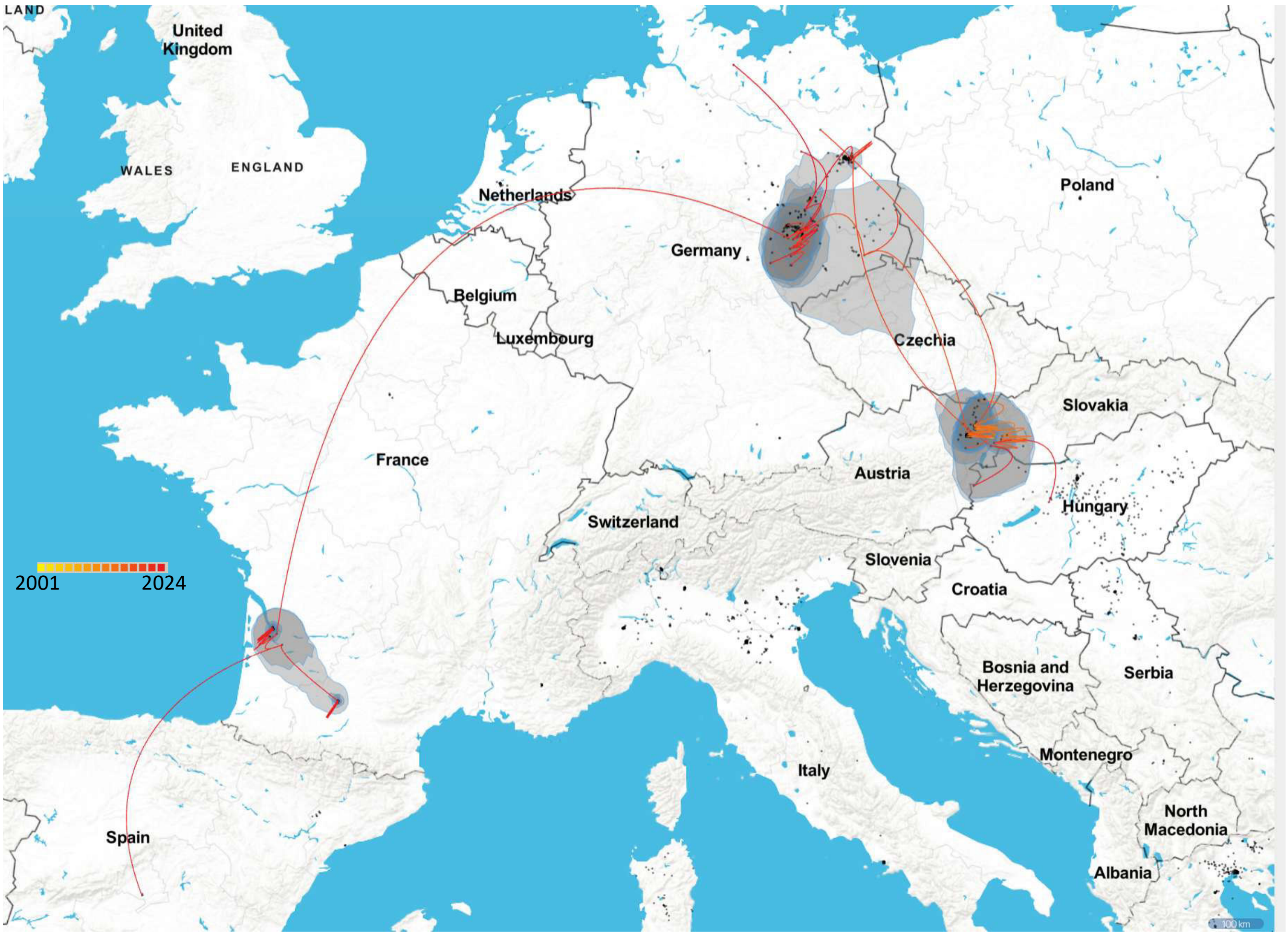
United Kingdom
WALES
ENGLAND
Netherlands
Belgium
Luxembourg
Germany
Poland
Czechia
Slovakia
Austria
Hungary
France
Switzerland
Slovenia
Croatia
Bosnia and Herzegovina
Serbia
Montenegro
North Macedonia
Albania
Italy
Spain
2001
2024

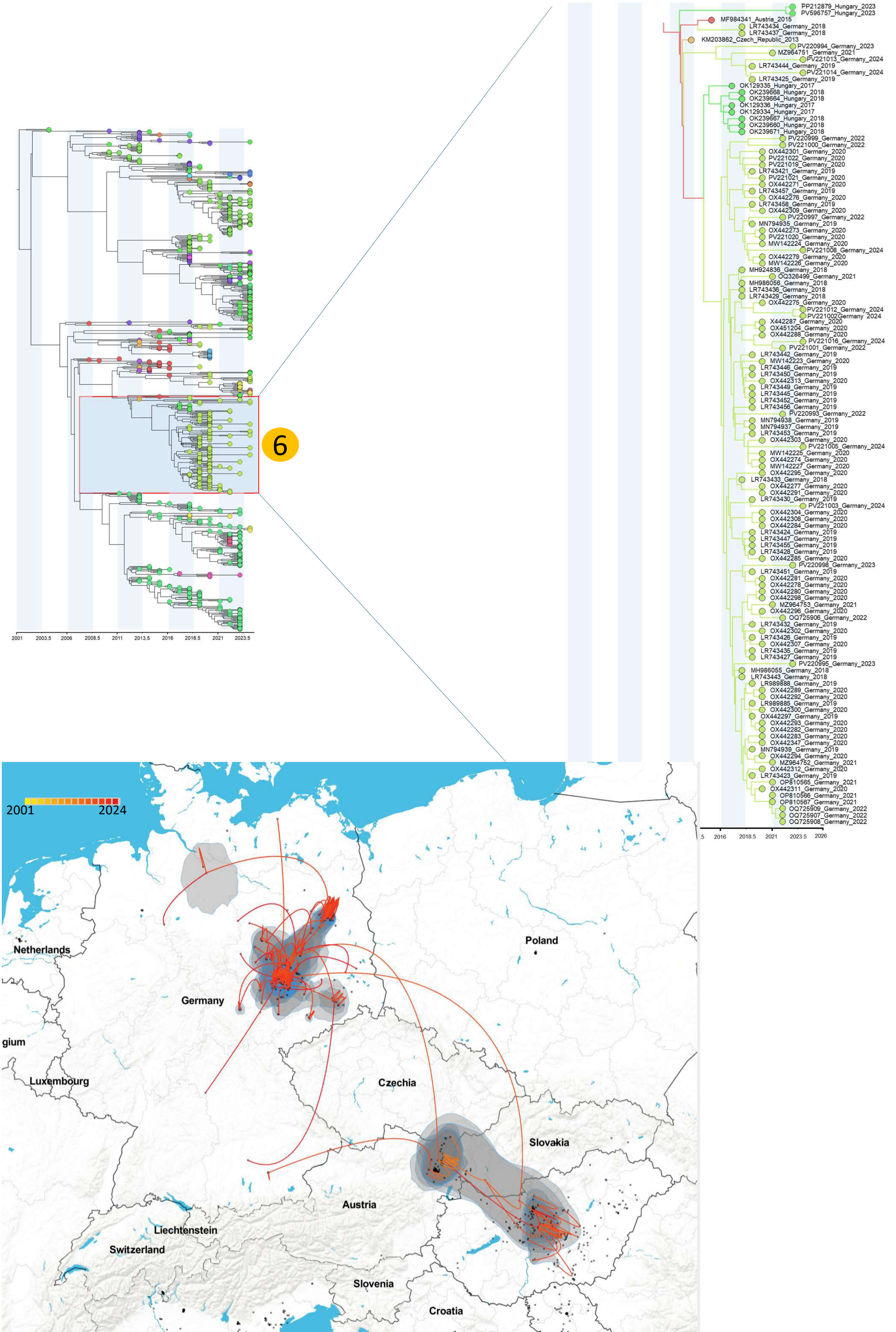
2001
2003.5
2006
2008.5
2011
2013.5
2016
2018.5
2021
2023.5
6
PP212879_Hungary_2023
PV596757_Hungary_2023
MF984341_Austria_2015
LR743434_Germany_2018
LR743437_Germany_2018
KM203862_Czech_Republic_2013
PV220994_Germany_2023
MZ964751_Germany_2021
PV221013_Germany_2024
LR743444_Germany_2019
PV221014_Germany_2024
LR743425_Germany_2019
OK129335_Hungary_2017
OK239668_Hungary_2018
OK239664_Hungary_2018
OK129336_Hungary_2017
OK129334_Hungary_2017
OK239667_Hungary_2018
OK239660_Hungary_2018
OK239671_Hungary_2018
PV220999_Germany_2022
PV221000_Germany_2022
OX442301_Germany_2020
PV221022_Germany_2020
PV221019_Germany_2020
LR743421_Germany_2019
PV221021_Germany_2020
OX442271_Germany_2020
LR743457_Germany_2019
OX442276_Germany_2020
LR743458_Germany_2019
OX442309_Germany_2020
PV220997_Germany_2022
MN794935_Germany_2019
OX442273_Germany_2020
PV221020_Germany_2020
MW142224_Germany_2020
PV221008_Germany_2024
OX442279_Germany_2020
MW142226_Germany_2020
MH924836_Germany_2018
OQ326499_Germany_2021
MH986056_Germany_2018
LR743436_Germany_2018
LR743429_Germany_2018
OX442275_Germany_2020
PV221012_Germany_2024
PV221002Germany_2024
X442287_Germany_2020
OX451204_Germany_2020
OX442288_Germany_2020
PV221016_Germany_2024
PV221001_Germany_2022
LR743442_Germany_2019
MW142223_Germany_2020
LR743446_Germany_2019
LR743450_Germany_2019
OX442313_Germany_2020
LR743449_Germany_2019
LR743445_Germany_2019
LR743452_Germany_2019
LR743456_Germany_2019
PV220993_Germany_2022
MN794938_Germany_2019
MN794937_Germany_2019
LR743453_Germany_2019
OX442303_Germany_2020
PV221005_Germany_2024
MW142225_Germany_2020
OX442274_Germany_2020
MW142227_Germany_2020
OX442295_Germany_2020
LR743433_Germany_2018
OX442277_Germany_2020
OX442291_Germany_2020
LR743430_Germany_2019
PV221003_Germany_2024
OX442304_Germany_2020
OX442308_Germany_2020
OX442284_Germany_2020
LR743424_Germany_2019
LR743447_Germany_2019
LR743455_Germany_2019
LR743428_Germany_2019
OX442285_Germany_2020
PV220998_Germany_2023
LR743451_Germany_2019
OX442281_Germany_2020
OX442278_Germany_2020
OX442280_Germany_2020
OX442298_Germany_2020
MZ964753_Germany_2021
OX442296_Germany_2020
OQ725906_Germany_2022
LR743432_Germany_2019
OX442302_Germany_2020
LR743426_Germany_2019
OX442307_Germany_2020
LR743435_Germany_2019
LR743427_Germany_2019
PV220995_Germany_2023
MH986055_Germany_2018
LR743443_Germany_2018
LR989888_Germany_2019
OX442289_Germany_2020
OX442292_Germany_2020
LR989885_Germany_2019
OX442300_Germany_2020
OX442297_Germany_2019
OX442293_Germany_2020
OX442282_Germany_2020
OX442283_Germany_2020
OX442347_Germany_2020
MN794939_Germany_2019
OX442294_Germany_2020
MZ964752_Germany_2021
OX442312_Germany_2020
LR743423_Germany_2019
OP810565_Germany_2021
OX442311_Germany_2020
OP810566_Germany_2021
OP810567_Germany_2021
OQ725909_Germany_2022
OQ725907_Germany_2022
OQ725908_Germany_2022
2016
2018.5
2021
2023.5
2026
2001
2024
Netherlands
Germany
gium
Luxembourg
Poland
Czechia
Slovakia
Austria
Liechtenstein
Switzerland
Slovenia
Croatia
